# Biokinetics of Protein Degrading *Clostridium cadaveris *and *Clostridium sporogenes *in Batch and Continuous Mode of Operations

**DOI:** 10.4014/jmb.1908.08054

**Published:** 2020-01-17

**Authors:** Taewoan Koo, Md Abu Hanifa Jannat, Seokhwan Hwang

**Affiliations:** Division of Environmental Science and Engineering, Pohang University of Science and Technology (POSTECH), Pohang 37673, Republic of Korea

**Keywords:** Protein degradation, *Clostridium cadaveris*, *Clostridium sporogenes*, quantitative real-time PCR, biokinetic analysis

## Abstract

A quantitative real-time polymerase chain reaction (QPCR) was applied to estimate biokinetic coefficients of *Clostridium cadaveris* and *Clostridium sporogenes,* which utilize protein as carbon source. Experimental data on changes in peptone concentration and 16S rRNA gene copy numbers of *C. cadaveris* and *C. sporogenes* were fitted to model. The fourth-order Runge-Kutta approximation with non-linear least squares analysis was employed to solve the ordinary differential equations to estimate biokinetic coefficients. The maximum specific growth rate (*μ_max_*), half-saturation concentration (*K_s_*), growth yield (*Y*), and decay coefficient (*K_d_*) of *C. cadaveris* and *C.sporogenes* were 0.73 ± 0.05 and 1.35 ± 0.32 h^-1^, 6.07 ± 1.52 and 5.67 ± 1.53 g/l, 2.25 ± 0.75 x 10^10^ and 7.92 ± 3.71 x 10^9^ copies/g, 0.002 ± 0.003 and 0.002 ± 0.001 h^-1^, respectively. The theoretical specific growth rate of *C. sporogenes* always exceeded that of *C. cadaveris* at peptone concentration higher than 3.62 g/l. When the influent peptone concentration was 5.0 g/l, the concentration of *C.cadaveris* gradually decreased to the steady value of 2.9 x 10^10^ copies/ml at 4 h Hydraulic retention time (HRT), which indicates a 67.1% reduction of the initial population, but the wash out occurred at HRTs of 1.9 and 3.2 h. The 16S rRNA gene copy numbers of *C. sporogenes* gradually decreased to steady values ranging from 1.1 x 10^10^ to 2.9 x 10^10^ copies/ml. *C. sporogenes* species was predicted to wash out at an HRT of 1.6 h.

## Introduction

Anaerobic digestion provides a potential cost-effective solution for the treatment of high-strength organics such as food waste because of low sludge production and methane formation, which can be used as a fuel [1, 2]. The anaerobic digestion is a multi-stage biochemical process in which crude organics are hydrolyzed and fermented into intermediates (*i.e*., mostly volatile organic acids, H_2_/CO_2_), which are subsequently converted into methane, carbon dioxide, and microbial cell masses [3]. The stages are referred to as hydrolysis, acidogenesis, and methanogenesis, respectively. These stages involve various species of symbiotic microbes, which are classified into two groups: acidogenic bacteria and methanogenic archaea [4]. These two groups of microorganisms differ widely in their growth environment and biokinetics [5].

Because of different substrate utilization and growth kinetics between acidogens and methanogens, it has been proposed that optimization of each of these stages would enhance the overall rate of waste stabilization if the biphasic ecosystem could be maintained in separate digesters in a series; one for acid production and one for methane production. Two-phase processes with partial acidification of influent wastewater have advantages in organic loading rate and gas production over single-phase processes [6, 7]. Thus, it has also been suggested that overall process enhancement must be based on an understanding of the behavior of acidogens, especially the optimization and mathematical modeling of the acidogenic stage [7].

Proteins are one of various crude organic materials including food waste and sludge for anaerobic digestion. Despite the important contribution of carbon and nitrogen sources for microbial growth, a low degree of protein degradation is frequently observed in full-scale anaerobic digesters [8]. A specialized group of gram-positive microorganisms, *e.g*. proteolytic *Clostridia* class, plays a dominant role in protein catabolism in an anaerobic digestion [9]. *Clostridium* is one of the dominant bacterial genera found in full-scale anaerobic digesters treating food waste, sludge, and livestock excrement [10-12]. Genus *Clostridium* includes more than 100 species, which show a variety of metabolic abilities to convert amino acids to various organic acids [1]. This diversity also suggests that these species have an important function in acidogenesis of protein during anaerobic digestion [13]. *Clostridium sporogenes*, for example, is found to be a dominant acidogenic bacteria species in a full-scale digester treating food waste, recycling wastewater[14], and in a lab-scale anaerobic reactor treating peptone [15]. *Clostridium cadaveris* is also known to degrade protein and amino acids [16].

Process biokinetics play a central role in the development and operation of diverse biological wastewater processes including anaerobic digestion systems. The biokinetic information is fundamental for understanding the mechanism with respect to microbial growth and substrate utilization in engineered ecosystems. The evaluation of biokinetic coefficients (*i.e*., maximum growth rate, substrate affinity, growth yield, and microbial decay rate) along with its application forms a basis for design and control of various biological processes [17].

Our main objective with this study, therefore, was to estimate the biokinetic coefficients of *C. cadaveris* and *C. sporogenes*, which utilize protein as carbon source. The coefficients of maximum growth, half-saturation, yield, and decay were estimated in batch cultivation of the species. The behavior and dynamics of the two species in continuous mode of operations at different hydraulic retention times were also simulated using the coefficients estimated.

## Materials and Methods

### Microbial Species and Culture Conditions

*C. cadaveris* (KCTC 5420) and *C. sporogenes* (KCTC 5654) were obtained from the Korean Collection for Type Cultures (KCTC) and enriched in anaerobic serum bottles on Reinforced Clostridial Media (RCM). The inoculum was operated to keep active cell condition. Each cell was transferred at the exponential growth from inoculum. The carbon source with nutrients of medium for both species include: 5 g peptone, 0.5 g yeast extract, 2 g ammonium acetate, 1 g NaCl, 0.75 g K_2_HPO_4_, 0.75 g KH_2_PO_4_, 0.5 g cysteine HCl•H_2_O and 200 μl trace elements solution in 1 l of deionized water. The trace elements contain 5 g MgSO_4_•7H_2_O, 0.5 g MnSO_4_•H_2_O and 0.5 g FeSO_4_•7H_2_O in 1 l of deionized water.

The batch experiments were conducted anaerobically in the 120 mL serum bottles to cultivate *C. cadaveris* and *C. sporogenes*. The peptone was the only sole carbon source for both species. The initial ODs were less than 0.01 at 600 nm wavelength. All experiments were duplicated. The cultivation of each species was conducted in an incubator at 37°C (N-BIOTEK, Korea) and pH 5.8 using 1N NaOH. The variations in peptone concentrations were monitored using the modification of the Lowry protein assay using bovine serum albumin (BSA) as a standard [18, 19].

### DNA Extraction and Quantitative polymerase chain reaction (QPCR) Analysis

After batch experiments, samples were centrifuged at 5,000 g for 5 min, supernatant was discarded and refilled with deionized water twice. DNA extraction was performed by automated nucleic acid extractors (Magtration System 12GC, PSS CO., Japan) with Genomic kit supplied by manufacturer. The extracted DNAs were stored at -20°C.

The QPCR analysis to determine the 16S rRNA gene copy numbers of *C. cadaveris* and *C. sporogenes* was performed using a LightCycler 480 (Roche Diagnostics, Germany). The specific primer and probe sets used in this study for targeting 16S rRNA gene copy numbers of *C. cadaveris* and *C. sporogenes* were developed, where verification of specificities was performed both in silico and in vitro (Table 1) [13]. The mixture of QPCR was 20 ul: 2 μl of template DNA, 1 μl each of forward primer and of reverse primer (final concentration 500 nM), 1 μl of the Taqman probe (final concentration 100 nM), 5 μl of LightCycler Taqman Master Mix (Roche Diagnostics), and 10 μl of PCR-grade water.

### Evaluation of Biokinetic Coefficients and Modeling Continuous System

Variations in peptone concentration and 16S rRNA gene copy numbers of *C. cadaveris* and *C. sporogenes* in batch experiments were monitored and used to evaluate the biokinetic coefficients. Monod growth kinetics was used to describe the growth of *C. cadaveris* and *C. sporogenes*.


(1) $$\mu =\frac{\mu_{m}S}{K_{s}+S}$$

(2) $$\frac{dX_{i}}{dt}=X_{i}\left(\frac{\mu_{mi}S}{K_{si}+S}-k_{di}\right),i=1,2$$

(3) $$\frac{dS}{dt}=-\frac{1}{Y_{i}}X_{i}\left(\frac{\mu_{mi}S}{K_{si}+S}\right)$$

Where *μ* is the specific growth rate (h^-1^), *μ_max_* is the maximum specific growth rate coefficient (h^-1^), *K_s_* is the half-saturation coefficient (g/l), S is the substrate concentration (g/l), *X_i_* is the i species 16S rRNA gene copy numbers (copies/ml), *K_d_* is the decay coefficient (h^-1^), and *Y* is the growth yield (copies/g). The subscripts of *i* represent species *C. cadavaeris* (1) and *C. sporogenes* (2) in order, respectively.

The fourth-order Runge-Kutta approximation with non-linear least squares analysis was employed to solve the ordinary differential equations (*i.e*., Eqs. 2 and 3) [17, 20, 21]. Matlab/Simulink software was used to estimate the biokinetic coefficients [22].

The residual peptone concentration and 16S rRNA gene copy numbers of *C. cadaveris* and *C. sporogenes* during dynamic and steady state operations at different HRTs (hydraulic retention time) were predicted using the biokinetic coefficients. The mathematical expressions used were:


(4) $$\frac{dX_{j}}{dt}=X_{j}\left(\frac{\mu_{mj}S}{K_{sj}+S}-\frac{1}{\tau }-k_{dj}\right)$$

(5) $$\frac{dS}{dt}=\frac{\left(S_{i-}S\right)}{\tau }-\frac{1}{Y_{j}}X_{j}\left(\frac{\mu_{mj}S}{K_{sj}+S}\right)$$

(6) $$\theta_{w}=\frac{K_{sj}+S_{i}}{S_{i}\left(\mu_{mj}-k_{dj}\right)-K_{sj}k_{dj}}$$

Where X*j* represent species *C. cadavaeris* and *C. sporogenes* in order, Si is the influent peptone concentration (g/l), τ is the hydraulic retention time (h), and θ*w* is the hydraulic retention time leading washout (h).

Standard deviation of estimated parameters and correlation between each parameter pair were evaluated from computation of entry of covariance matrix (V), which was inverse of the Fisher information matrix (FIM) [22].


(7) $$FIM_{j}={\sum_{i=1}^{N_{data}}\left(\frac{\partial y}{\partial \theta }\left(t_{ji,}\varphi_{k}\right)\right)}^{T}\left(\frac{\partial y}{\partial \theta }\left(t_{ji,}\varphi_{k}\right)\right),j=1,2,3,4;k=1,2$$

(8) $$V_{j}=FIM_{j}^{-1}$$

Where Ndata is the total experiment data number, y is state variable (S, X_i_, X_j_), δy/δθ is the sensitive function, *φ*_k_ is the parameter vectors (*μ_mk_*, *K_sk_*, *Y_ik_*, *K_dk_*).

## Results and Discussion

### Performance and Biokinetic Estimation in Pure Culture

**Fig. 1:**
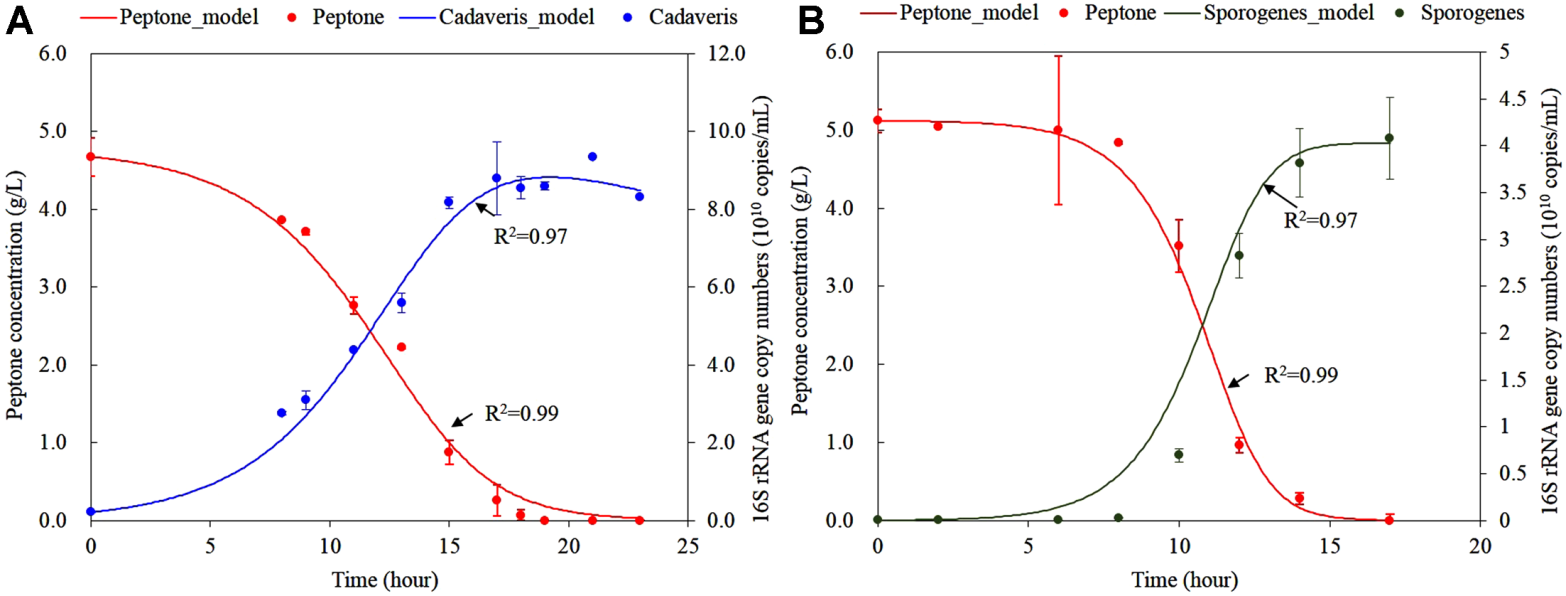
Peptone degradation and microbial concentrations. (**A**) Change in peptone concentration (solid red triangle, left y-axis), *C. cadaveris* concentration (solid blue circle, right y-axis) in pure culture. (**B**) Change in peptone concentration (solid red triangle, left y-axis), *C. sporogenes* concentration (solid dark green circle, right y-axis) in pure culture. *Points* observed value; *lines* predicted value.

Batch experiments were conducted until residual peptone concentration reached to zero and microbial concentration was stable in pure culture (Fig. 1). During the culture period, *C. cadaveris* and *C. sporogenes* consumed peptone completely within 19 and 17 h, respectively. The initial 16S rRNA gene copy numbers for *C. cadaveris* and *C. sporogenes* were 2.1 ± 0.02 × 10^9^ and 3.2 ± 0.02 × 10^8^ copies/ml, respectively. Those of *C. cadaveris* and *C. sporogenes* drastically increased and reached maximum concentration of 8.8 ± 0.01 and 4.1 ± 0.08 × 10^10^ copies/ml at 17 h, respectively.

The experimental observations of the residual peptone concentration and 16S rRNA gene copy numbers of *C. cadaveris* and *C. sporogenes* were fitted to the models (*i.e*., Eqs. 2 and 3) to evaluate biokinetic coefficients (Fig. 1). The models showed statistically significant fits with high R^2^ (>0.97) which implied that the obtained biokinetic coefficients were reliable.

The estimated biokinetic coefficients of two species were summarized in Table 2. The maximum specific growth rate of *C. sporogenes* (*i.e*., 1.35 h^-1^) was higher than that of *C. cadaveris* (*i.e*., 0.73 h^-1^). The maximum specific growth rate shows the maximum value when the available substrate concentration to the species is not growth limiting, and represents the steepest slope when microbial growth is in an exponential phase [17, 20]. The half-saturation coefficient, *K_s_*, of *C. sporogenes* (*i.e*., 5.67 g/l) was lower than that of *C. cadaveris* (*i.e*., 6.07 g/l). The *K_s_* is commonly used as an indicator of the substrate affinity of microbes. The lower half-saturation coefficient value, the higher the substrate affinity. The growth yield of *C. cadaveris* was 2.9 times higher than that of *C. sporogenes*, which indicated the 16S genes copy numbers of *C. cadaveris* increased nearly 3 times that of *C. sporogenes* at given unit amount of peptone. This meant the amount of sludge produced per unit amount of protein would likely be that much higher. Decay coefficient was 0.002 for both species showing no significant difference.

Standard deviation of each coefficient derived from uncertainty analysis (Table 2). The analysis was performed based on a 95% confidence interval. These results indicated the estimated parameters were practically identified and reliable. Correlation coefficients between parameter pairs were lower than 0.9, which was considered to be the threshold of high correlation (Table 3) [5].

### Competitive Specific Growth Rate of *C. cadaveris* and *C. sporogenes*

**Fig. 2:**
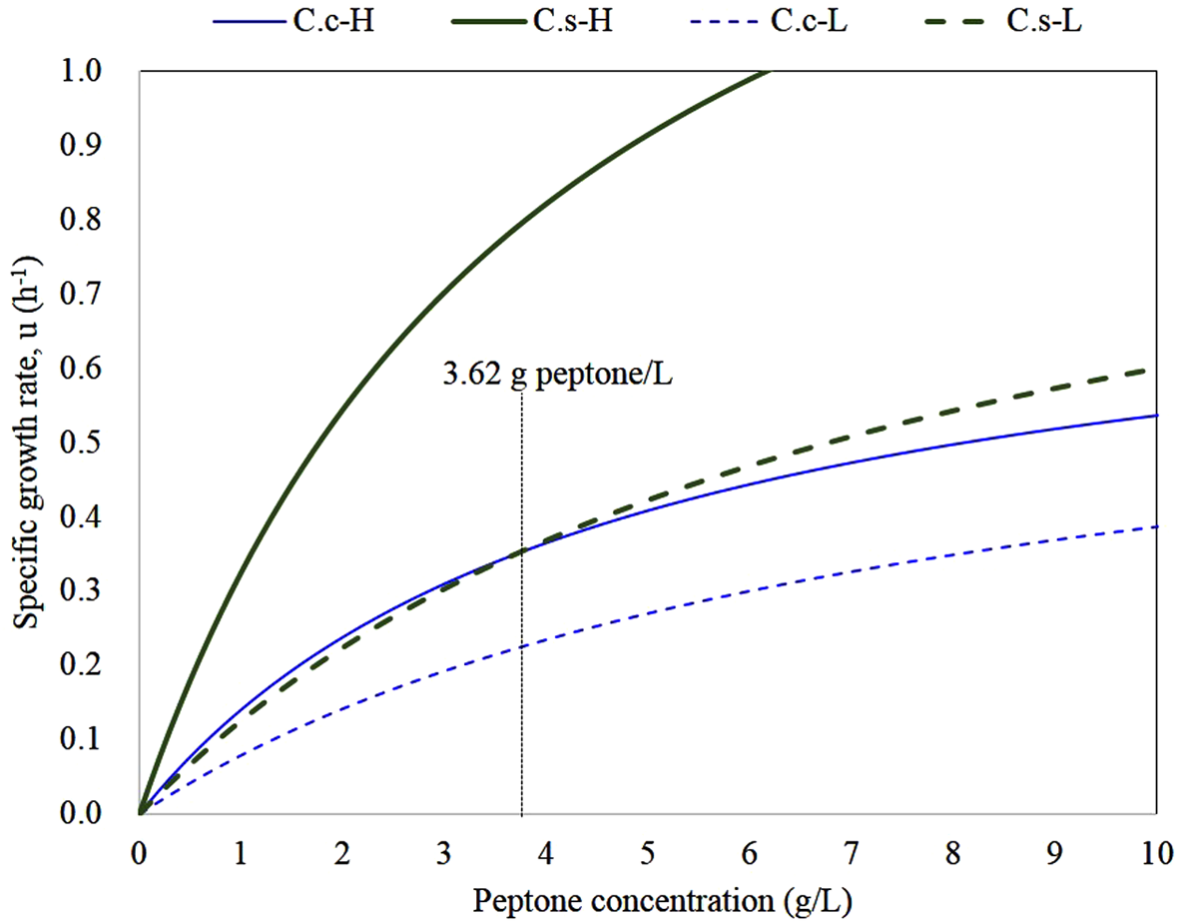
Hypothetical specific growth rate curve of *C. cadaveris* and *C. sporogenes* at peptone as sole carbon source. Solid and dotted blue thin lines are theoretical highest and lowest specific growth rate curves of *C. cadaveris* (*i.e*., C.c-H and C.c-L), respectively. Solid and dotted dark green thick lines are theoretical highest and lowest specific growth rate curves of *C. sporogenes* (*i.e*., C.s-H and C.s-L), respectively. The peptone concentration, where the minimum specific growth of *C. sporogenes* and the maximum specific growth of *C. cadaveris* rate are equal.

In order to compare the growth patterns of the two species considering 95% confidence interval, the hypothetical competitive specific growth rate relationships between *C. cadaveris* and *C. sporogenes* for peptone utilization were shown in Fig. 2. The maximum specific growth rate, *μ_m_*, and half-saturation concentration, *K_s_*, in the Monod equation derived from pure culture were used to simulate specific growth rate as a function of peptone concentration. The growth rate curves for C.c-H and C.s-H (Fig. 2) represented the theoretical maximum specific growth rate curves of *C. cadaveris* and *C. sporogenes*, which were calculated with the highest *μ_m_* (*i.e*., 0.78 and 1.67 h^-1^) and the lowest *K_s_* (*i.e*., 4.55 and 4.14 g/l) for the corresponding species, respectively. On the other hand, the theoretical minimum specific growth rate curves of *C. cadaveris* and *C. sporogenes* were computed using the lowest *μ_m_* (*i.e*., 0.68 and 1.03 h^-1^) and the highest *K_s_* (*i.e*., 7.59 and 7.20 g/l), which were C.c-L and C.s-L, respectively.

It is evident that the specific growth rate of *C. sporogenes* always exceeds that of *C. cadaveris* at peptone concentration higher than 3.62 g/l. The growth rate of *C. cadaveris* was also seen to slightly exceed *C. sporogenes* at peptone concentration higher than 3.62 g/l. These results also suggest that the two species may compete for protein utilization at lower than 3.62 g/l. Likewise, a population shift between the two species would occur at low protein concentration.

### Simulation of Continuous Operation at Different HRTs

**Fig. 3:**
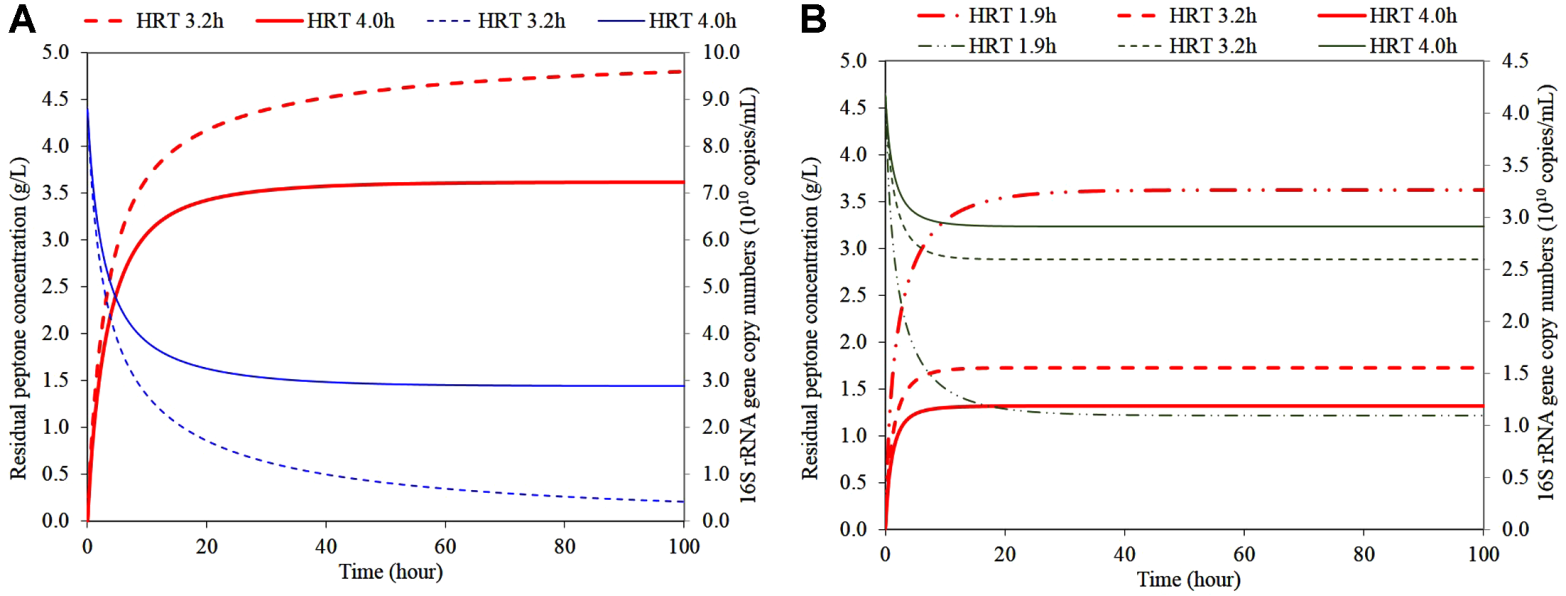
Temporal changes in residual peptone and microbial concentrations. (**A**) Simulation results in residual peptone concentration (red thick line, left y-axis), *C. cadaveris* concentration (blue thin line, right y-axis) at different HRTs in continuous condition in pure culture. (**B**) Simulation results in residual peptone concentration (red thick line, left y-axis), *C. sporogenes* concentration (dark green thin line, right y-axis) at different HRTs in continuous condition in pure culture. An influent peptone concentration was applied at 5.0 g/l. The initial microbial concentrations of *C. cadaveris* and *C. sporogenes* were 8.8 × 10^10^ and 4.2 × 10^10^ copies/ml, respectively.

In order to investigate the microbial behaviors of the two species at around 3.62 g peptone/l in continuous mode, an additional simulation using Eqs. 4 and 5 with the kinetic coefficients was performed. Fig. 3 represents the changes in residual peptone and microbial concentrations in dynamic regions, including steady state at different HRTs. The steady state was assumed when the gene copy numbers and residual peptone concentration did not vary by more than 5%.

The influent peptone concentration was assumed to be 5.0 g/l, close to the start-up conditions as the batch culture. The simulation was initiated with the initial 16S rRNA gene copy numbers of 8.8 × 10^10^ and 4.2 × 10^10^ for *C. cadaveris* and *C. sporogenes*, the same as the maximum values in the batch experiment, respectively. Fig. 3 showed the temporal changes in residual peptone and microbial concentrations of each species at dynamic and steady states predicted at different HRTs. Table 4 summarized the process performances predicted at different HRTs. The HRTs to give the residual peptone concentrations of 3.62 g/l for *C. cadaveris* and *C. sporogenes* were 4.0 and 1.9 h, respectively.

The concentration of *C. cadaveris* gradually decreased to the steady value of 2.9 × 10^10^ at 4 h HRT, which indicated 67.1% of the initial population reduction. A washout of *C. cadaveris*, resulting in a continuous increase in residual peptone concentration to the input concentration of 5.0 g/l, was expected at 3.2 h HRT. This was the calculated washout point of 3.2 h using the Eq. (6). This gradual washout of *C. cadaveris* was shown in Fig. 3A.

Fig. 3B shows temporal changes in the residual peptone concentration and microbial 16S rRNA gene copy numbers of *C. sporogenes*, and depicts steady states at different HRTs with respect to the variations in *C. cadaveris* populations. The residual peptone concentrations increased and thereafter remained steady. The expected treatment efficiency ranged from 73.6% to 27.6% at the assigned HRTs. Based on these simulations, the estimated residual peptone concentration would be lower than 3.62 g/l at HRTs longer than 1.9 h, which was well below washout HRT of *C. cadaveris*. The 16S rRNA gene copy numbers of *C. sporogenes* gradually decreased to steady values ranging from 1.1 × 10^10^ to 2.9 × 10^10^ copies/ml. *C. sporogenes* species was predicted to washout at an HRT of 1.6 h using the Eq. (6).

### Comparison of Performance between Pure and Mixed Culture

**Fig. 4:**
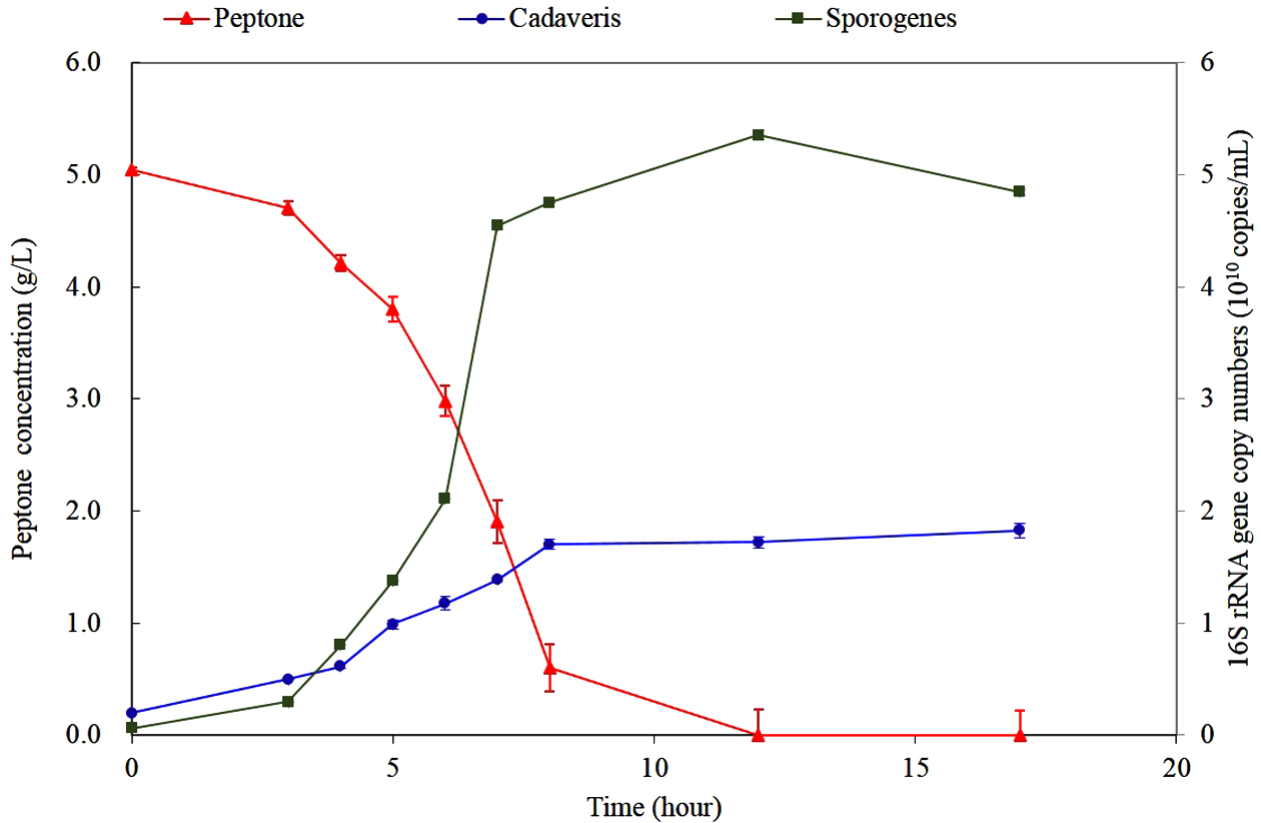
Change in peptone concentration (solid red triangle, left y-axis), *C. cadaveris* concentration (solid blue circle, right y-axis), and *C. sporogenes* concentration (solid dark green square, right y-axis) in mixed culture.

Fig. 4 indicated that the trend of peptone consumption and microbial growth in mixed culture was different from those observed in the pure cultures. The peptone was completely consumed within 12 h, which was 5 and 7 h faster than the peptone consumption time of *C. cadaveris* and *C. sporogenes* in pure culture, respectively. The initial 16S rRNA gene copy numbers for *C. cadaveris* and *C. sporogenes* were 1.9 ± 0.5 × 10^9^ copies/ml and 6.2 ± 0.04 × 10^9^ copies/ml, respectively. The 16S rRNA gene copy numbers for *C. cadaveris* and *C. sporogenes* increased and reached maximum concentration of 1.8 ± 0.3 and 5.4 ± 0.2 × 10^10^ copies/ml at 17 and 12 h, respectively. The maximum 16S rRNA gene copy number of *C. sporogenes* was 2.9 times higher than that of *C. cadaveris*. The maximum 16S rRNA gene copy number of *C. cadaveris* in mixed culture (1.8 × 10^10^ copies/ml) was 20.8% of that in pure culture (8.8 × 10^10^ copies/ml), which indicated the *C. cadaveris* population increased by nearly one fourth in mixed culture. On the other hand, the maximum 16S rRNA gene copy number of *C. sporogenes* in mixed culture (5.4 × 10^10^ copies/ml) was 130.5% of that in pure culture (4.1 × 10^10^ copies/ml), which indicated *C. sporogenes* outcompetes *C. cadaveris* in the mixed culture. The time to reach the maximum 16S rRNA gene copy number of *C.* *sporogenes* (*i.e*., 12 h) in mixed culture was 5 h faster than that in pure culture (*i.e*., 17 h). The time to reach the maximum 16S rRNA gene copy number of *C. cadaveris* (*i.e*., 17 h) in mixed culture was the same as that in pure culture (*i.e*., 17 h).

The first-order reaction expression was used to estimate the rate coefficient of substrate utilization, *k*, (h^-1^). Those in *C. cadaveris* and *C. sporogenes* in pure cultures were 0.24 and 0.32 h^-1^, respectively, while that in mixed culture increased to 0.33 h^-1^. This suggested peptone degradation rate increased when the two species coexisted.

The difference between pure and mixed cultures was whether other species exist, and all other experimental conditions were the same and controlled. In other words, the change in the rate of substrate consumption and the microbial growth rate in pure culture and mixed culture could be attributed to the presence of other species, which implied that interspecific interaction occurred. These interspecific interactions could affect the growth rate of each species [23, 24]. The majority of interspecies interaction mechanisms are made by biochemical molecules secreted by microorganisms without physical contact [25]. Some species in the *Clostridium* genus were already known to produce and secrete oligopeptides as chemical signals for interspecies interactions [26]. These results suggested that a new model describing interspecific interaction was necessary in environments where different species shared the same substrate.

QPCR analysis was successfully applied to the biokinetic analysis of peptone utilization and 16S rRNA gene concentration of *C. cadaveris* and *C. sporogenes* to derive the biokinetic coefficients of each species. All coefficients were reliable and identifiable. The maximum specific growth rate (*μ_max_*), half-saturation concentration (*K_s_*), growth yield (*Y*), and decay coefficient (*K_d_*) of *C. cadaveris* and *C.sporogenes* were 0.73 ± 0.05 and 1.35 ± 0.32 h^-1^, 6.07 ± 1.52 and 5.67 ± 1.53 g/l, 2.25 ± 0.75 × 10^10^ and 7.92 ± 3.71 × 10^9^ copies/g, 0.002 ± 0.003 and 0.002 ± 0.001 h^-1^, respectively. Using the derived biokinetic coefficients, the specific growth rate simulation predicted the outcompeting of *C. sporogenes*. Modeling with Continuous stirred-tank reactor (CSTR) system also predicted the different process performance of each species at the same HRTs. The changes in the rate of peptone consumption and microbial growth were demonstrated by comparing the pure and mixed cultures of *C. cadaveris* and *C. sporogenes* utilizing a protein substrate simultaneously. The biokinetic coefficients derived from this study can be used to predict acidogenic behavior and process efficiency in protein-rich wastewater. Although biokinetics is essential for practical engineering, these coefficients are not of constant value and depend on environment conditions. Therefore, the biokinetic coefficients derived from this study can be used to predict similar environments.

## Figures and Tables

**Table 1:** Characteristics of the specific primer and probe sets targeting 16s rRNA gene copy numbers of the *Clostridium cadaveris* and *Clostridium sporogenes*.

Name	Function1)	Target species	Sequence (5’ 3’)	Species numbering	Tm2) (°C)	Amplicon size (bp)
Cadav-791F	F primer	*Clostridium*	CTAGGTGTAGGGGTTTCGA	791-809	59.3	344
Cadav-992T	TM probe	*cadaveris*	TTCGGGAGCAGGAACACAGGTG	992-1015	68.3	
Cadav-1116R	R primer		GTTAACCACGGCAGTCTAG	1116-1134	59.6	
Sporo-157F	F primer	*Clostridium*	TTAATACCGCATAACATAAGAGAA	157-180	58	280
Sporo-207T	TM probe	*sporogenes*	ATTGCTTTGAGATGGACCCGCG	207-228	66.9	
Sporo-418R	R primer		CCAGAAAACAGGGCTTTAC	418-436	58.1	

^1)^TM, TaqMan.

^2)^Calculated with the nearest-neighbor thermodynamic values method using the DNAMAN program (500 nM of primer, 100 nM of probe, and 100 mM of salt concentration)

**Table 2:** Estimated biokinetic coefficients and 95% confidence intervals.

Parameter	Clostridium cadaveris	Clostridium sporogenes
*µ_m_* (h^-1^)	0.73 ± 0.05	1.35 ± 0.32
*K_s_* (g/l)	6.07 ± 1.52	5.67 ± 1.53
*Y* (copies/g)	2.25 ± 0.75 × 10^10^	7.92 ± 3.71 × 10^9^
*K_d_* (h^-1^)	0.002 ± 0.003	0.002 ± 0.001

**Table 3:** Correlation matrix of each species.

*C. cadaveris*					*C. sporogenes*				

	*μ_m_*	*K_s_*	Y	*k_d_*		*μ_m_*	*K_s_*	Y	*k_d_*
*μ_m_*	1	0.47	0.77	0.73	*μ_m_*	1	0.59	0.71	0.57
*K_s_*	0.47	1	0.54	0.75	*K_s_*	0.59	1	0.76	0.48
Y	0.77	0.54	1	0.79	Y	0.71	0.76	1	0.28
*k_d_*	0.73	0.75	0.79	1	*k_d_*	0.57	0.48	0.28	1

**Table 4:** Predicted process performance of steady-state condition in pure culture at various HRTs.

		*C. cadaveris*			*C. sporogenes*		

HRT (h)	Loading rate (g peptone/l/h)	Residual peptone concentration (g/l)	Removal efficiency (%)	Microbial concentration (10^10^ copies/ml)	Residual peptone concentration (g/l)	Removal efficiency (%)	Microbial concentration (10^10^ copies/ml)
1.9	2.6	5.0	washout	washout	3.6	27.6	1.1
3.2	1.6	5.0	washout	washout	1.7	65.5	2.6
4.0	1.3	3.6	27.6	2.9	1.3	73.6	2.9

Initial conditions of the simulation: Influent concentration: 5.0 g peptone/l; Both species: 0.0 × 10^10^ copies/ml; Initial concentration: 0 g peptone/l; *C. cadaveris*: 8.8 × 10^10^ copies/ml; C. sporogenes: 4.2 × 10^10^ copies/ml
